# Adiponectin in eutrophic and obese children as a biomarker to predict metabolic syndrome and each of its components

**DOI:** 10.1186/1471-2458-13-88

**Published:** 2013-01-30

**Authors:** Miguel Klünder-Klünder, Samuel Flores-Huerta, Rebeca García-Macedo, Jesús Peralta-Romero, Miguel Cruz

**Affiliations:** 1 Community Health Research Department, Hospital Infantil de México Federico Gómez, Ministry of Health (SSA), Mexico City, Mexico; 2 Medical Research Unit in Biochemistry, UMAE Bernardo Sepúlveda, Centro Médico Nacional Siglo XXI, IMSS, Mexico City, Mexico

**Keywords:** Obesity, Adiponectin, Child, Insulin resistance, Metabolic syndrome, Biomarker

## Abstract

**Background:**

Obesity is associated with the rise of noncommunicable diseases worldwide. The pathophysiology behind this disease involves the increase of adipose tissue, being inversely related to adiponectin, but directly related to insulin resistance and metabolic syndrome (MetS). Therefore, this study aimed to determine the relationship between adiponectin levels with each component of MetS in eutrophic and obese Mexican children.

**Methods:**

A cross sectional study was conducted in 190 school-age children classified as obese and 196 classified as eutrophic. Adiponectin, glucose, insulin, high density lipoprotein cholesterol (HDL-C) and triglycerides were determined from a fasting blood sample. Height, weight, waist circumference, systolic and diastolic blood pressures (BP) were measured; MetS was evaluated with the IDF definition. The study groups were divided according to tertiles of adiponectin, using the higher concentration as a reference. Linear regression analysis was used to assess the association between adiponectin and components of the MetS. Finally, stepwise forward multiple logistic regression analysis controlling for age, gender, basal HOMA-IR values and BMI was performed to determine the odds ratio of developing MetS according to adiponectin tertiles.

**Results:**

Anthropometric and metabolic measurements were statistically different between eutrophic and obese children with and without MetS (P <0.001). The prevalence of MetS in obese populations was 13%. Adiponectin concentrations were 15.5 ± 6.1, 12.0 ± 4.8, 12.4 ± 4.9 and 9.4 ± 2.8 μg/mL for eutrophic and obese subjects, obese without MetS, and obese with MetS, respectively (P <0.001). Obese children with low values of adiponectin exhibited a higher frequency of MetS components: abdominal obesity, 49%; high systolic BP, 3%; high diastolic BP, 2%; impaired fasting glucose, 17%; hypertriglyceridemia, 31%; and low HDL-C values, 42%. Adjusted odds ratio of presenting MetS according to adiponectin categories was 10.9 (95% CI 2.05; 48.16) when the first tertile was compared with the third.

**Conclusion:**

In this sample of eutrophic and obese Mexican children we found that adiponectin concentrations and MetS components have an inversely proportional relationship, which supports the idea that this hormone could be a biomarker for identifying individuals with risk of developing MetS.

## Background

During the last three decades, obesity has emerged worldwide as an epidemic affecting all ages and socioeconomic levels. In Mexico, the prevalence of obesity in school-age children has an upward trend [1]. The Mexican National Health and Nutrition Survey 2006 reported an increase of 77% and 47% in boys and girls respectively, between 1999 and 2006 [2].The childhood obesity epidemic has led to a parallel rise in the prevalence of pediatric forms of chronic illnesses such as type 2 diabetes (T2D) and high blood pressure (BP), which in the recent past were typically adult diseases [3,4].

Despite the fact that the classification of metabolic syndrome (MetS) has several limitations, for instance the use of categorical criteria in each of its components [5,6], it is a useful clinical tool to identify individuals with risk of developing diseases associated with obesity.

Currently, the pathophysiology behind this syndrome begins with the fact that the adipose tissue, visceral and ectopic, functions as an endocrine gland producing an imbalance between pro and anti-inflammatory cytokines. Pro-inflammatory cytokines such as TNF-α, IL-6, IL-1β, and PAI-1, increase; while adiponectin, an anti-inflammatory cytokine, decreases [7]. This imbalance elicits low-degree systemic inflammation associated with insulin resistance [8]. Owing to that after weight reduction, adiponectin increases, improving insulin sensitivity throughout the body [9-12], its plasmatic concentration has been proposed as a candidate biomarker to identify metabolic alterations including those of MetS [13-15]. However, in children this adiponectin role still remains controversial [16,17].

Therefore, the aim of this study was to determine the relationship between plasmatic adiponectin levels and MetS components in eutrophic and obese children, and to assess whether this hormone could be a biomarker for MetS in Mexican children.

## Methods

This epidemiological study was conducted from November 2007 to December 2010 in nine middle-class schools of Mexico City. Prior to the study, ethical clearance was obtained by both the ethics and research institutional boards of the Hospital Infantil de México “Federico Gómez” and school authorities; likewise, written informed consent was obtained from the participants and their parents in each of the two stages of the study. The first stage aimed to identify obese school-age children. For this purpose and following international anthropometric guidelines [18], a trained team of nurses measured children’s weight and height without shoes and wearing light clothing. Weight was taken using a digital scale (Seca, Hamburg, Germany) to the nearest 0.1 kg; height was measured using a Seca 225 stadiometer to the nearest 0.1 cm. This screening was performed in 1,441 children between 6 and 12 years of age, and eutrophic or obese status was assessed using BMI percentiles according to the 2000 Centers for Disease Control and Prevention reference (Atlanta, GA, USA) [19].

Eutrophic status was considered when BMI was between 25th and 75th percentile and obese when the BMI was >95th percentile for the child´s age and gender. Because the purpose of the study was assessing if plasmatic adiponectin levels categorized in tertiles were able to identify a certain profile as healthy or at risk, the sample was designed to have contrasting nutritional status groups, either normal or obese. In order to fulfill the study´s aims, individuals between normal and obese (BMI >75th pc < 95th pc) or between normal and malnourished (BMI < 25th pc) were purposefully excluded.

Of all the participants, 286 were obese (19.9%), 20 of them did not meet the inclusion criteria and 59 did not accept to participate. In 190 out of 207 individuals complete blood samples were collected. According to our criteria, there were 524 eutrophic children of which 250 were invited to participate and 209 accepted. In 196, whole blood samples were drawn. Those who self-reported as having an acute infectious disease, suffering from chronic illness such as allergies and autoimmune diseases that could alter cytokines patterns, or those who were participating in a weight reduction program were excluded.

In the second stage, BP was obtained by auscultatory method using a sphygmomanometer (ALPK2, Tokyo, Japan) with appropriate cuff size for arm length, following North American guidelines issued in 2004 [20]. Four BP readings were taken for each participant on the right arm in a sitting position, resting 1 minute between each measurement. The mean of the last three readings was considered the final level of BP. Waist circumference (WC) was measured at the midpoint between the lowest rib and the iliac crest after a normal exhalation with children in the standing position. A fasting blood sample was used to determine insulin by chemiluminescence immunoassay (IMMULITE 2000, Euro, DPC, Llanberis, UK), glucose, total cholesterol, low-density lipoprotein cholesterol (LDL-C), high-density lipoprotein cholesterol (HDL-C), and triglycerides (ILAB 300, Instrumentation Laboratory, Barcelona, Spain). Intra and inter assay coefficients of variation (CV) values were <5%. Total adiponectin was determined by ELISA method (Human Adiponectin ELISA Kit, Millipore, St. Charles, MO, USA), in which the mean of the minimum detectable concentration was 0.78 ng/mL. Intra- and inter assay CV were <7.4 and <8.4 %, respectively. All analyses were performed during the 2–5 days following the blood sample extraction. HOMA-IR was obtained by the following equation: [fasting glucose (mg/dL) x fasting insulin (μU/mL)/405]. A 3.4 HOMA-IR value was the cut-off point to accept IR, which corresponds to the 90^th^ percentile in a population of healthy children [21].

### Definition of MetS

MetS was defined according to guidelines of the International Diabetes Federation (IDF) [22], with the exception of BP in which case the criterion used was according to the North American Task Force guidelines [20]. Therefore, MetS was diagnosed by abdominal obesity (WC ≥90^th^ percentile for child’s age, gender and ethnic origin) [23,24] and the presence of two or more other clinical features: triglycerides ≥150 mg/dL; HDL-C ≤40 mg/dL, BP systolic and/or diastolic ≥90^th^ percentile for child’s age, gender and height; fasting glucose ≥100 mg/dL.

### Statistical analysis

Anthropometric and metabolic data were tested for normal distribution using skewness and kurtosis. Comparisons between continuous variables and groups were performed with Student t test or Mann–Whitney U test according to data distribution. In order to assess the effect of adiponectin on each of the MetS components, insulin and HOMA-IR, this hormone was categorized in tertiles, and obese individuals were reclassified to those with or without MetS. Differences in metabolic features between adiponectin tertile categories were obtained using linear regression analysis. The prevalence of each component of MetS was obtained within each of the adiponectin tertiles. To assess the odds of developing MetS, these were compared in whole population according to the previously mentioned adiponectin tertile categories using logistic regression analysis and adjusted by BMI and sex and age. P <0.05 was accepted as statistically significant. Data were processed with STATA, SE v.11.0, and EPIINFO 3.3.2 according to the 2000 CDC reference [19].

## Results

Anthropometric features, blood pressure, metabolic profile and adiponectin concentrations of eutrophic and obese children with or without MetS are shown in Table 1. Age range of the participants was 6–12 years and the prevalence of MetS in the obese population was ~13%. BMI was different between eutrophic and obese participants and also between obese children with or without MetS (P =0.004). BP (systolic and diastolic) values and percentiles were different between eutrophic and obese children (P <0.001) and also between children with or without MetS (P <0.05).

**Table 1:** Anthropometric, clinical and metabolic characteristics in eutrophic and obese children with and without MetS

**Characteristics**	**Eutrophic**	**Obese**		**Obese**		
				**Without MetS**	**With MetS**	
	**n** = **196**	**n** = **190**		**n** = **166**	**n** = **24**	
	**Mean** ± **SD**	**Mean** ± **SD**	**P**^**d**^	**Mean** ± **SD**	**Mean** ± **SD**	**P**^**e**^
Age (years)	9.4 ± 1.8	9.6 ± 1.8	0.368	9.6 ± 1.9	9.4 ± 1.4	0.623
Anthropometric measurements						
WC (cm)	57.8 ± 4.9	79.0 ± 9.1	<0.001	78.3 ± 9.2	83.5 ± 6.5	0.004
BMI (kg/m^2^)	16.4 ± 1.3	24.5 ± 2.7	<0.001	24.3 ± 2.8	25.8 ± 2.5	0.009
BMI percentile^a^	46.1 ± 18.2	97.4 ± 1.3	<0.001	97.2 ± 1.3	98.3 ± 0.9	<0.001
Blood pressure (mm/Hg)						
SBP	88.6 ± 7.8	97.8 ± 8.8	<0.001	97.1 ± 8.2	101.3 ± 10.8	0.028
SBP percentile^b^	17.3 ± 18.2	33.8 ± 22.9	<0.001	32.0 ± 21.1	43.2 ± 30.6	0.037
Diastolic	55.8 ± 7.1	62.2 ± 7.6	<0.001	61.4 ± 7.2	65.9 ± 7.9	0.009
DBP percentile^b^	37.7 ± 20.9	53.6 ± 21.5	<0.001	51.7 ± 20.5	63.5 ± 22.3	0.021
Metabolic parameters*						
Glucose (mg/dL)	90.0 (57.0-14.0)	90.0 (58.0-108.0)	0.794	90.0 (58.0-108.0)	94.0 (54.0-107.0)	0.090
Insulin (mU/mL)	4.3 (1.9-19.5)	9.3 (2.0-68.0)	<0.001	9.1 (2.0-55.2)	14.1 (3.8-68.0)	<0.001
HOMA-IR^c^	1.0 (0.3-5.0)	2.0 (0.4-13.9)	<0.001	2.0 (0.4-13.9)	3.5 (0.7-11.2)	<0.001
TGs (mg/dL)	72.0 (22.5-193.5)	106.5 (30.0-366.0)	<0.001	104.0 (30.0-366.0)	166.0 (72.9-255.0)	<0.001
HDL-C (mg/dL)	53.0 (21.0-110.0)	44.0 (9.0-82.0)	<0.001	45.0 (20.0-82.0)	36.0 (9.0-51.0)	<0.001
Inflammatory*						
Adiponectin (μg/mL)	14.5 (3.9-38.8)	11.5 (1.4-27.5)	<0.001	12.2 (1.4-27.5)	8.4 (4.2-15.3)	0.003

^a^According to CDC 2000.

^b^According to The National High Blood Pressure Education Program.

^c^HOMA-IR: [(fasting glucose (mg/dL))(fasting insulin (μU/mL))/ 405)].

^d^ t test p value for means or Mann Whitney U test for medians: eutrophic vs. eutrophic obese, ^e^Obese without MetS vs. obese with MetS.

*Median (min-max).

MetS, metabolic syndrome; WC, waist circumference; BMI, body mass index; SBP, systolic blood pressure; DBP, diastolic blood pressure; TGs, triglycerides; HDL-C, high-density lipoprotein cholesterol; MetS, metabolic syndrome.

In terms of metabolic variables, obese children with MetS exhibited higher values of these variables than obese children without MetS and eutrophic children. Glucose values were not different between groups, but insulin concentrations increased significantly in obese children and especially in obese children with MetS (P <0.001). HOMA-IR values within the obese group with MetS were higher than those for the other groups (P <0.001). A similar relationship was observed with triglycerides (P <0.001). Another parameter that differed was HDL-C, in which case the obese group with MetS showed the lowest values (P <0.001). Adiponectin levels in eutrophic children were higher compared with the obese group (P < 0.001). Interestingly, lower concentrations of adiponectin were observed in the obese with MetS compared with obese without MetS (P =0.003).

**Figure 1:**
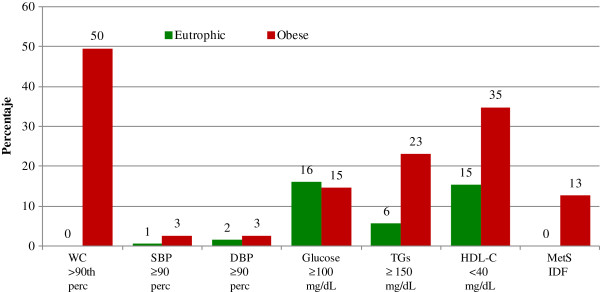
**Prevalence of metabolic syndrome components by nutritional status.** WC, waist circumference; SBP, systolic blood pressure; DBP, diastolic blood pressure; TGs, triglycerides; HDL-C, high-density lipoprotein cholesterol.

**Figure 2:**
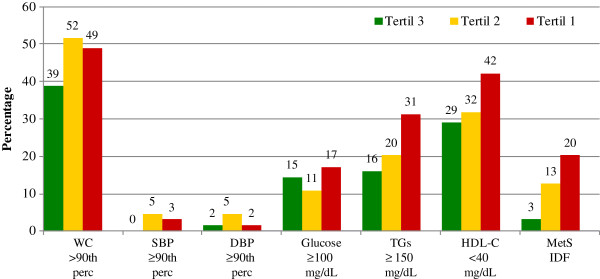
**Prevalence of metabolic syndrome components in obese children**, **according to adiponectin tertile.** WC, waist circumference; SBP, systolic blood pressure; DBP, diastolic blood pressure; TGs, triglycerides; HDL-C, high-density lipoprotein cholesterol.

Figures 1 and 2, show the prevalence of MetS and each of its altered components, according to nutritional status and adiponectin concentration. Figure 1 demonstrates that none of the eutrophic children had MetS showing a low prevalence in fasting glucose, HDL-C, and triglyceride alterations. Likewise, none of them had altered waist circumference and virtually no impairment in BP was shown. In contrast, 13% of the obese children had MetS. The most prevalent alterations in the obese group were waist circumference (50%), HDL-C (35%), triglycerides (23%), and fasting glucose (15%). Children with altered BP were very few in both groups.

Figure 2 shows that prevalence of MetS components increase as adiponectin concentrations decrease in obese children. When adiponectin values were in the first tertile, 31% of these children had hypertrigliceridemia and 42% had low HDL-C values, compared to children in the third tertile who presented 16% and 29%, respectively.

Table 2 shows a trend of increasing insulin and HOMA-IR when adiponectin values decreased in eutrophic and obese children (p < 0.05). Also, in obese children the appearance of the components of MetS was higher when adiponectin values were in the low tertile (P <0.05).

**Table 2:** Insulin resistance and MetS components according to level of adiponectin, in eutrophic and obese children and adolescents

	**Adiponectin tertile(values)**					
	**Eutrophic subjects(n** **=** **196)**			**Obese subjects(n** **=** **190)**		
	**3**	**2**	**1**	**3**	**2**	**1**
Outcome variables	(17.6-38.8)	(12.6-17.5)	(3.9-12.5)	(13.4-27.5)	(9.6-13.3)	(1.4-9.5)
Insulin resistance						
Fasting insulin (μm/mL)						
Coefficient	Referent	0.9	1.1	Referent	3.3	3.4
95% CI	-	−0.1; 1.9	0.1; 2.1	-	−0.1; 6.5	0.2; 6.7
P	-	0.083	0.032	-	0.053	0.039
HOMA-IR						
Coefficient	Referent	0.2	0.3	Referent	0.6	0.8
95% CI	-	−0.1; 0.4	0.1; 0.5	-	−0.2; 1.3	0.1; 1.6
P	-	0.123	0.035	-	0.126	0.022
Components of MetS						
WC (cm)						
Coefficient	Referent	0.9	3.1	Referent	4.9	5.9
95% CI	-	−0.6; 2.4	1.6; 4.6	-	2.1; 7.7	3.1; 8.7
P	-	0.229	<0.001	-	0.001	<0.001
Blood pressure (mmHg)						
SBP						
Coefficient	Referent	0.8	1.9	Referent	3.8	4.5
95% CI	-	−1.8; 3.5	−0.8; 4.5	-	0.9; 6.7	1.6; 7.3
P	-	0.538	0.168	-	0.010	0.002
DBP						
Coefficient	Referent	1.6	2.5	Referent	1.7	0.9
95% CI	-	−0.9;4.0	0.1; 5.0	-	−1.0; 4.3	−1.8; 3.5
P	-	0.213	0.043	-	0.585	0.738
Glucose (mg/dL)						
Coefficient	Referent	0.1	0.5	Referent	−0.4	3.9
95% CI	-	−3.6; 3.9	−3.2; 4.3	-	−3.9; 3.1	−0.4; 7.4
P	-	0.962	0.789	-	0.140	0.029
TGs (mg/dL)						
Coefficient	Referent	−2.3	4.8	Referent	14.4	27.3
95% CI	-	−13.5; 8.9	−6.2; 15.9	-	−8.0; 36.8	4.9; 49.6
P	-	0.931	0.391	-	0.206	0.017
HDL-C (mg/dL)						
Coefficient	Referent	2.2	−0.3	Referent	−1.3	−4.1
95% CI	-	−2.4; 6.8	−5.2; 4.5	-	−5.3; 2.6	−8.1; 0.1
P	-	0.351	0.894	-	0.511	0.048

Values obtained through linear regression and adjusted for gender and body mass index percentile.

Regression coefficients are change in outcome variables per tertile change in adiponectin concentration.

MetS, metabolic syndrome; WC, waist circumference; SBP, systolic blood pressure; DBP, diastolic blood pressure; TGs, triglycerides; HDL-C, high-density lipoprotein cholesterol.

In obese children, WC and systolic BP increased when adiponectin decreased to the second tertile (P = 0.001; P =0.010). Additionally, when adiponectin decreased to the low tertile, WC and systolic BP significantly increased; 5.9 cm in WC (P <0.001), and 4.5 mmHg in systolic blood pressure (P =0.002). Glucose increased 3.9 mg/dL (P =0.029). Triglycerides increased 27.3 mg/dL (P = 0.017), whereas HDL-C decreased 4.1 mg/dL (P = 0.048).

Table 3 shows how the odds of developing MetS are higher when adiponectin levels are lower (adjusted for gender, age, BMI and HOMA-IR). When adiponectin is in the first tertile, the odds ratio of developing MetS is 10.3 compared with the third tertile (95% CI 2.05; 48.16).

**Table 3:** Odds ratio of developing MetS according to adiponectin tertile value

**Variable**	**OR**	**95**% **CI**	**P**
Gender (female)	0.8	0.28; 2.46	0.737
Age (years)	0.8	0.57; 1.12	0.200
BMI (percentile)	2.0	1.20; 3.39	0.008
HOMA-IR	1.4	1.10; 1.73	0.006
Adiponectin tertile*			
3	1	-	-
2	4.8	0.84; 26.83	0.078
1	10.3	2.05; 48.16	0.005

Values obtained through logistic regression analysis, the final model included whole study population (obese and eutrophic children) adjusted for BMI (body mass index) percentile, gender, age and HOMA-IR values. *Tertiles used are according to nutritional status of each child as shown in Table 2.

## Discussion

Our study found that adiponectin levels are associated with MetS as a whole and with each of its components, mainly in obese children.

Considering the age of participants, some of them could be in the pubertal stage, especially girls who can enter this phase up to two years earlier than boys [25]. Unfortunately, the pubertal stage was not measured in the study, and we cannot discriminate the potential influence of sexual hormones on changes in adiponectin. However, in studies in which the pubertal stage has been controlled, results regarding the association between adiponectin and MetS are controversial. Some of them show that pubertal stage mediates the association between adiponectin and MetS [26,27], while others performed in Latin-American children did not find this observation [14,28]. In the present study, adiponectin concentration between boys and girls were similar with values of 12.8 and 13.3 μg/mL, respectively (p = 0.362).

Concerning the prevalence of 13% of MetS in obese children reported herein, and in order to properly locate this figure in relationship to others, we compared this prevalence with other studies in which the IDF definition was applied. In doing so, we found that the prevalence of MetS in obese children has a wide variation; in Greek children is 7.7%, in Colombian is 11.7% [27,29], in Chinese and Spanish obese children is 27.6 and 19.6% respectively [30,31], and in another study on Mexican obese children the prevalence was 20% [32]. Notwithstanding the same IDF definition of MetS, variation could be explained by the differences in the environment in which the children live.

It is noteworthy that independent of nutritional status of the children, when their adiponectin levels are in the lowest tertile all the components of MetS worsen; WC, diastolic BP, glucose, and triglycerides increase and HDL-C values decrease. On the other hand, when adiponectin levels are in the high tertiles, each component of MetS improves even in obese children, although it is not possible to say that these individuals are metabolically healthy.

A similar phenomenon but with less magnitude is observed in eutrophic children. Nowadays, we do not have a plausible way of explaining why adiponectin in eutrophic children decreases because they are not exposed to the inflammatory environment of obesity; however, eutrophic children with adiponectin concentrations in the low tertile have significantly higher values of HOMA IR, WC and diastolic BP than those in the high tertile. To our knowledge, the present work is the first to show that adiponectin also influences metabolic changes in eutrophic children, even after adjustment of adiponectin levels by BMI and gender.

In a recent bariatric surgery study that was made up of morbidly obese patients, the association between adiponectin genes and adiponectin expression was studied in eight patients with T2D and four control patients [33]. Adiponectin production was downregulated in obese patients but upregulated once obesity was reduced. Its production rose to levels close to the control group, suggesting that adiponectin expression is dysregulated with obesity. The versatile replenishment of this adipokine with weight loss supports the importance of using this cytokine as a biomarker in prevention programs as well as in treatment and control of diseases, especially T2D.

Although there are reports in which adiponectin has not been shown to be a useful biomarker for monitoring changes observed in MetS [16,17], evidence exists for postulating this cytokine as a biomarker of MetS and T2D in obese children and adolescents [13,14,34,35]. However, the molecular mechanisms that establish the relationship between adiponectin and metabolic derangements observed in clinical and epidemiological studies, have not been fully elucidated [36]. The most plausible explanation is that obesity, mainly visceral, is a condition in which there is an inflammatory state characterized by dysregulated production of adipokines in which anti-inflammatory agents such as adiponectin decrease while pro-inflammatory cytokines such TNFα and IL-6, IL 1β increase. Unfortunately pro- inflammatory cytokines were not measured in order to demonstrate their predominance, although at present, there is practically no discussion about the negative association between central obesity and adiponectin levels [37,38].

The use of adiponectin as a marker for MetS has already been shown in a 3-year prospective study where adiponectin levels in Korean children predicted the development of the MetS [26].

In addition to aforementioned limitations, it is necessary to highlight that this study is cross-sectional in design, which only permits associations but not causality to be established among different variables. Lastly, because the study was performed in an ethnically homogenous population in Mexico City its results may not apply to other populations. Despite these limitations, the negative association between adiponectin and the components of MetS suggest that these children have an imbalance in inflammatory adipokines.

## Conclusion

In this sample of eutrophic and obese Mexican children, an inversely proportional relationship was found between adiponectin concentrations and MetS components, which supports the idea that this hormone could be a possible biomarker to identify individuals with risk of developing MetS.

## Competing interests

The authors declare that they have no competing interests.

## Authors' contributions

MKK participated in the design and coordination of the study, performed the statistical analysis, and revised the manuscript critically. SFH participated in the conception and design of the research question as well as in fieldwork supervision. RGM analyzed blood samples and revised the manuscript critically. JPR Obtained data and analyzed blood samples. All authors were involved in drafting the manuscript. All of them read and approved the final version of the manuscript.

## Pre-publication history

The pre-publication history for this paper can be accessed here:

http://www.biomedcentral.com/1471-2458/13/88/prepub
